# Evolving antimicrobial resistance patterns in group B streptococci: A five-year study in a high-risk pregnancy referral center in northeastern Brazil

**DOI:** 10.1590/0037-8682-0259-2025

**Published:** 2026-03-06

**Authors:** Jorhanna Isabelle Araújo de Brito Gomes, Suellen Bernardo de Queiroz, Carlos Gabriel Andrade Barbosa, Larissa Rodrigues Santos Silva, Patrícia Urquiza Lundgren, Eduardo Sergio Soares Sousa, Eloiza Helena Campana, Vinicius Pietta Perez

**Affiliations:** 1 Universidade Federal da Paraíba, Centro de Ciências da Saúde, Núcleo de Medicina Tropical, João Pessoa, PB, Brasil.; 2 Hospital Universitário Lauro Wanderley, Laboratório de Análises Clínicas, João Pessoa, PB, Brasil.; 3 Universidade Federal da Paraíba, Centro de Ciências Médicas, João Pessoa, PB, Brasil.; 4 Universidade Federal do Rio Grande do Sul, Instituto de Ciências Básicas da Saúde, Departamento de Microbiologia, Imunologia e Parasitologia, Porto Alegre, RS, Brasil.

**Keywords:** Streptococcus agalactiae, Antimicrobial resistance, Intrapartum antibiotic prophylaxis, Pregnant women, Macrolides, Clindamycin

## Abstract

**Background:**

*Streptococcus agalactiae* (commonly referred to as group B streptococci [GBS]) is a leading cause of neonatal infection. Surveillance of colonization in pregnant women and the use of intrapartum antibiotic prophylaxis (IAP) are the primary strategies for preventing early-onset GBS disease. The increasing rate of antibiotic resistance among GBS isolates is a concern for the effectiveness of IAP. Our study aimed to evaluate the prevalence of GBS colonization and characterize antimicrobial resistance patterns over a five-year period in a high-risk pregnancy referral center in Northeastern Brazil.

**Methods:**

This study was conducted from 2020 to 2024 and included pregnant women at 35-37-week gestation. GBS isolates from anal-vaginal swabs were identified and tested for susceptibility to penicillin or ampicillin, clindamycin, erythromycin, levofloxacin, and tetracycline. Isolates stored from 2021 to 2024 were further analyzed for the resistance genes *mef(A), erm(A/TR), erm(B), tet(M),* and *tet(O).*

**Results:**

Of 1469 anal-vaginal samples, the overall GBS colonization rate was 12%. All isolates were susceptible to either penicillin or ampicillin. The respective resistance rates for erythromycin, clindamycin, levofloxacin, and tetracycline were 23.6%, 9.3%, 5.4%, and 82.3%. The main determinant among macrolide-resistant isolates was *mef(A)*, and *tet(M)* was the most frequent tetracycline resistance gene. Furthermore, we found that erythromycin resistance increased consistently over the five years, signaling a potential impact on clindamycin efficacy due to *erm* genes.

**Conclusions:**

Beta-lactams (penicillin and ampicillin) remained effective for IAP in northeastern Brazil during the study period. However, high and increasing resistance to other antibiotic classes reinforces the need for maternal GBS surveillance and ongoing antimicrobial resistance monitoring.

## INTRODUCTION

*Streptococcus agalactiae,* commonly referred to as group B streptococci (GBS), is a commensal colonizer of the human genitourinary and gastrointestinal tracts. It is also a significant pathogen associated with neonatal diseases, including sepsis, pneumonia, and meningitis, which often result in neurodevelopmental impairment in survivors^1^.

Neonatal disease can manifest as early onset infection (EOI) in the first week of life and is associated with vertical transmission during childbirth. To prevent EOI, maternal screening for GBS colonization between 36 and 37 weeks of gestation with administration of intrapartum antibiotic prophylaxis (IAP) to colonized mothers is recommended. Late-onset infection (LOI) occurs between the first week and three months of age, and transmission can be associated with the maternal microbiota or environmental sources^2^.

Although beta-lactams remain the first-line IAP agents, variations in GBS susceptibility to these antibiotics have been reported worldwide, particularly reduced susceptibility has been sporadically reported^3^. Resistance to macrolides has prompted revisions to IAP protocols, and susceptibility to clindamycin, the second-line agent, must be confirmed. Furthermore, the use of fluoroquinolones to treat urinary tract infections in many regions is associated with increased resistance, and tetracycline resistance is a common characteristic of GBS strains^4^. 

To optimize IAP protocols and guide treatment strategies, continuous surveillance of GBS antibiotic resistance is therefore essential. In northeastern Brazil, data regarding GBS epidemiology are lacking, and antenatal screening for GBS is recommended only for high-risk pregnancies in Brazil^5^. Our study aimed to investigate the GBS colonization rate, antimicrobial susceptibility profile, and resistance genotypes to macrolides and tetracyclines in pregnant women at a referral center in northeastern Brazil over a period of five years.

## METHODS

### Study design and samples

This observational single-center cross-sectional study was conducted between January 2020 and December 2024 at a gynecology and obstetrics referral center for high-risk pregnancies in João Pessoa, PB, northeast Brazil. Pregnant women at gestational age of 35-37 weeks who visited the high-risk antenatal care unit and underwent GBS screening were included in the study. Additionally, isolates identified as GBS and obtained from anal-vaginal swab screenings conducted during the years 2021 and 2024 were transferred into Brain Heart Infusion broth supplemented with 10% glycerol and stored at −20°C. This study was approved by the Hospital Universitário Lauro Wanderley Ethical Committee (Code Number: 3.155.051).

### GBS screening, bacterial identification and susceptibility testing

Sample collection was performed using rayon swabs, which were initially inserted approximately 2 cm into the vaginal canal and then gently introduced into the anal sphincter. The swabs were stored in Stuart transport medium and subsequently transferred to tubes containing Todd-Hewitt broth, followed by incubation at 35 ± 1°C for 8 h. After incubation, tubes showing visible growth (turbidity) were inoculated onto blood agar plates and incubated at 35 ± 1°C in a CO_2_ enriched atmosphere for 24-48 h. Beta-hemolytic colonies were selected, and catalase, Christie-Atkins-Munch-Petersen (CAMP), and L-pyrrolidonyl-beta-naphtylamide (PYR) tests were performed for identification. Antimicrobial susceptibility testing was performed for penicillin or ampicillin, clindamycin, erythromycin, levofloxacin, and tetracycline using the disk diffusion method, according to the Brazilian Committee on Antimicrobial Susceptibility Testing (BrCAST) guidelines. These guidelines were based on the European Committee on Antimicrobial Susceptibility Testing (EUCAST) standards.

### Detection of resistance genes by polymerase chain reaction (PCR)

PCR assays for the presence of resistance genes and detection of the *cfb* gene were performed on the stored isolates. One colony from each isolate (2021 and 2024) was transferred to a 10% solution of Chelex-100, vortexed vigorously, and incubated in a dry bath at 95°C for 30 min. The solution was then centrifuged at 4,000 RPM for 30 s, and the supernatant was transferred to a microtube for PCR. All amplification reactions were performed with a total volume of 20 µL using MiniAmp thermal cycler. Two microliters of DNA were added to PCR reaction mix containing 0.20 µM of each primer^6^ ^-^ ^8^ (Table 1), 0.2 mM of each dNTP, 1 U Taq polymerase, 2 mM of MgCl_2_, and reaction buffer. The cycling parameters were as follows: 95°C for 1 min, 35 cycles of 95°C for 1 min, annealing for 1 min (Table 1), extension at 72°C for 1 min, and final extension at 72°C for 5 min. The PCR product was run on 2% agarose gel in Tris Acetate EDTA (TAE) buffer, stained with a UV nucleic acid stain, and visualized with a UV trans-illuminator. The results were evaluated according to the expected amplicon sizes (Table 1).

**TABLE 1:** Oligonucleotide primer sequences for polymerase chain reaction (PCR) assays to detect *cfb* gene of *Streptococcus agalactiae* and resistance genes.

Target	Sequence (5’-3’)	Size (bp)	Annealing temperature (^o^C)	Reference
*cfb*	TTTCACCAGCTGTATTAGAAGTA GTTCCCTGAACATTATCTTTGAT	153	62	Kerdsin et al. 2017^6^
*erm(A/TR)*	AACTTGTGGAAATGAGTCAACGG CAGAATCTACATTAGGCTTAGGG	375	60	Pérez-Trallero et al. 2007^7^
*erm(B)*	ATTGGAACAGGTAAAGGGCG GAACATCTGTGGTATGGCG	442	60	Pérez-Trallero et al. 2007^7^
*mef(A)*	AGTATCATTAATCACTAGTGC TTCTTCTGGTACTAAAAGTGG	345	50	Pérez-Trallero et al. 2007^7^
*tet(M)*	GTGGAGTACTACATTTACGAG GAAGCGGATCACTATCTGAG	359	50	Poyart et al. 2003^8^
*tet(O)*	GCGGAACATTGCATTTGAGGG CTCTATGGACAACCCGACAGAAG	538	50	Poyart et al. 2003^8^

### Statistical analysis

Data are expressed as absolute and relative frequencies. Chi-square or Fisher’s exact test was used to explore and compare frequencies across different study years. Additionally, binary outcomes (GBS colonization and antibiotic resistance) were subjected to logistic regression analysis to investigate linear trends over the study period. All statistical analyses were conducted using SPSS (version 20.0) for Mac OS (IBM Corporation, Armonk, NY, USA). A 95% confidence interval (CI) was calculated, and the level of significance was set at 0.05.

## RESULTS

A total of 1469 anal-vaginal swabs from pregnant women were analyzed. The prevalence of anal-vaginal GBS colonization in our population was 12%, ranging from 17.8% in 2021 to 5.0% in 2023 (P < 0.001), the numbers across the five years are detailed in Table 2. The logistic regression showed a significant trend of reduced colonization over the years (odds ratio [OR] = 0.855; 95% CI = 0.763-0.958; P = 0.007).

**TABLE 2:** Prevalence of *Streptococcus agalactiae* colonization in 1,469 high-risk pregnant women (35-37-week gestation) in João Pessoa, PB, Brazil (2020-2024).

Year	Colonized		Non-colonized		Total
	n	%	n	%	
2020	37	14.2	224	85.8	261
2021	61	17.8	282	82.2	343
2022	26	9.7	242	90.3	268
2023	15	5.0	288	95.0	303
2024	38	12.9	256	87.1	294

**FIGURE 1:**
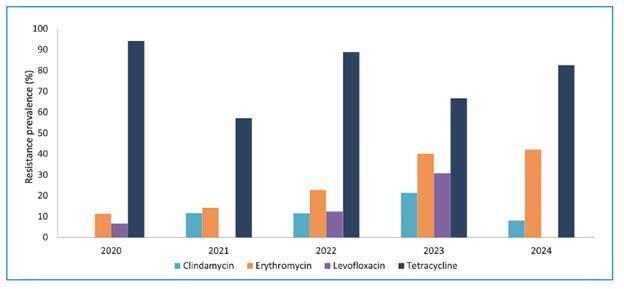
Antibiotic resistance rates and trends among *Streptococcus agalactiae* isolates from high-risk pregnant women (35-37-week gestation) in João Pessoa, PB, Brazil (2020-2024).

Of the 177 isolates obtained from colonized pregnant women, all were susceptible to beta-lactams (penicillin and/or ampicillin). Resistance rates to clindamycin, erythromycin, levofloxacin, and tetracyclines are shown in Figure 1. The overall resistance rate against tetracyclines was 82.3% (51/62): 94.1% (16/17) in 2020, 57.1% (4/7) in 2021, 88.9% (8/9) in 2022, 66.7% (4/6) in 2023, and 82.6% (19/23) in 2024 (P = 0.202). The overall resistance rate against erythromycin was 23.6% (39/165): 11.4% (4/35) in 2020, 14.2% (10/60) in 2021, 22.7% (5/22) in 2022, 40% (4/10) in 2023, and 42.1% (16/38) in 2024 (P = 0.010). Clindamycin resistance rate was 9.3% overall (16/172): 0% (0/35) in 2020, 11.7% (7/60) in 2021, 11.5% (3/26) in 2022, 21.4% (3/14) in 2023, and 8.1% (3/37) in 2024 (P = 0.085). The overall resistance rate against levofloxacin was 5.4% (6/111): 6.7% (1/15) in 2020, 0% (0/49) in 2021, 12.5% (1/8) in 2022, 30.8% (4/13) in 2023, and 0% (0/26) in 2024 (P < 0.001). Ten isolates, 6.1% (10/165), presented susceptibility only with increased exposure to erythromycin: 2.9% (1/35) in 2020 and 15% (9/60) in 2021. 

A significant increase in erythromycin resistance between 2020 to 2024 was observed (OR = 1.556; 95% CI = 1.211-2.000; P = 0.001). However, logistic regression analysis showed no statistically significant increase in resistance over the five-year period to clindamycin (OR = 1.212; 95% CI = 0.854-1.720; P = 0.282), levofloxacin (OR = 1.261; 95% CI = 0.717-2.217; P = 0.422), and tetracycline (OR = 0.883; 95% CI = 0.591-1.319; P = 0.543). 

To determine the resistance genotypes against macrolides and tetracyclines during the study period, anal-vaginal isolates from two years (2021 and 2024) were selected to evaluate the presence of *mef(A), erm(A/TR), erm(B), tet(M),* and *tet(O)* genes. Of the 61 and 38 isolates collected in 2021 and 2024, respectively, 36 and 21 isolates were stored and analyzed using PCR. The prevalence rates were as follows: for *mef(A)* 19.3% (19.4% in 2021 and 19.0% in 2024), for *erm(B)* 8.8% (8.3% in 2021 and 9.5% in 2024), for *erm(A/TR)* 3.5% (2.8% in 2021 and 4.8% in 2024), for *tet(M)* 82.4% (80.5% in 2021 and 85.7% in 2024), and for *tet(O)* 5.3% (5.5% in 2021 and 4.8% in 2024). Table 3 presents the percentages for each genotype among the strains.

**TABLE 3:** Genotypes of resistant *Streptococcus agalactiae* obtained from high-risk pregnant women (35-37-week gestation) in João Pessoa, PB, Brazil.

Antibiotic	2021		2024		Total	
	n	%	n	%	n	%
**Macrolides**						
*mef(A)*	7^a^	70.0^a^	4	66.7	11	68.6^a^
*erm(A/TR)*	1	10.0	1^b^	16.7^b^	2	12.5^b^
*erm(B)*	3^a^	30.0^a^	2^b^	33.3^b^	5	31.3^ab^
**Tetracyclines**						
*tet(M)*	28	93.3	18	94.7	46	93.9
*tet(O)*	2	6.7	1	5.3	3	6.1

^a^ isolate HU75 harbored *mef* and *erm(B)*. ^b^ isolate HU206 carried *erm(B)* and *erm(A/TR)*.

## DISCUSSION

Our five-year observational study provides valuable data on the prevalence and antimicrobial resistance of GBS among pregnant women in João Pessoa, Brazil. Maternal colonization rates vary regionally; a meta-analysis estimated a global prevalence of 18%, ranging from 11% to 35%, and a prevalence of 15.7% in South America^9^. The overall colonization rate of 12% in our population aligns with previous studies in Brazil, which reported a prevalence of 4.2-28.4% in the last decade, mainly in southern and southeastern Brazil^10^. We observed significant year-to-year variation, with rates peaking at 17.8% and declining to 5%, which was also observed in a single-center study in Rio de Janeiro, Brazil, from 13.8% in 2019 to 5.3% in 2020, suggesting that behavioral and healthcare changes during the COVID-19 pandemic (March 2020 to May 2023) may have affected colonization rates^5^. Notably, in our population, the peak prevalence was observed in 2021, followed by a significant reduction in 2023, potentially reflecting behavioral changes in the local population during the post-pandemic period.

Culture methods for GBS screening present low sensitivity, estimated at 54-70%, which is mainly due to non-hemolytic strains that cannot be identified using blood agar. The use of chromogenic agar and Nucleic Acid Amplification Tests (NAATs) has increased GBS detection from 18.8% to 24.4% and from 15% to 31.5%, respectively^11^ ^-^ ^12^. In Brazil, owing to the need for specialized infrastructure and high costs, the use of NAATs and chromogenic agar for GBS screening is restricted, which is likely related to the reduced prevalence observed in the low- and middle-income regions of Brazil when compared with data from southern and southeastern Brazil.

All isolates in our study were susceptible to beta-lactam antibiotics, underscoring the continued efficacy of penicillin and ampicillin for empirical IAP. However, erythromycin is no longer recommended for IAP^13^. This is due to the increasing resistance of GBS to macrolides, mediated by the efflux pump encoded by *mef(A)* gene or erythromycin methylases (*erm* genes). 

We also observed a consistent and statistically significant increase in erythromycin resistance, from 11.4% in 2020 to 42.1% in 2024. A recent report from southeastern Brazil revealed a trend of macrolide resistance (from 17% to 37%) during the pandemic period. This increase in prevalence was mostly associated with *mef(A)* gene dissemination (from 66.7% to 80.0%) and may be related to the indiscriminate use of azithromycin during the pandemic^14^. However, we did not observe any changes in the prevalence of resistance genes among isolates from 2021 to 2024, and *mef(A)* was the most frequent resistance gene during both the periods (70% versus 66.7%), followed by *erm(B)* and *erm(A/TR)*.

The *mef(A)* gene is related to the M phenotype (resistance only to macrolides) and has only slight effect on GBS disease. However, *erm* genes confer the MLSb phenotype (combined resistance to macrolides-lincosamides-streptogramin) and mediate resistance to clindamycin, a suitable second-line IAP and an important therapy for invasive GBS disease. Resistance to clindamycin is also associated with an infrequent L phenotype (resistance only to lincosamides), driven by *lnuB*, *lsaC*, and *lsaE* genes^3^. 

Our data show a concerning clindamycin resistance from 0% in 2020 to a peak of 21.4% in 2023, and an overall resistance rate of 9.3%. Conversely, no significant increase in clindamycin resistance was observed during the study period. In Brazil, clindamycin resistance over the last ten years (2-6.9%)^4^ ^,^ ^5^ ^,^ ^14^ ^-^ ^15^ has remained below the global estimate of 29.3% reported in a recent meta-analysis^16^. Furthermore, studies have reported a decreasing trend or stabilization of resistance to clindamycin in Brazil throughout the pandemic period^5^ ^,^ ^14^. Across the five years of our study, 93.7% of clindamycin-resistant isolates presented the MLSb phenotype related to the *erm* genes, and the L phenotype was observed only in one isolate in 2023. Indeed, a previous study using whole-genome sequencing in our setting did not report the L phenotype; however, it revealed the presence of *lnuB* and *lsaE* in two GBS strains harboring a fragment sequence related to a plasmid from *Enterococcus faecium* ^4^. In summary, our data suggest that the increasing prevalence of erythromycin-resistant strains, some of which harbor *erm* genes, could be the primary cause of clindamycin resistance in our population, and further studies should evaluate this important issue.

Fluoroquinolones, such as levofloxacin, are not indicated for IAP; however, they are frequently prescribed for urinary tract infections. Levofloxacin is included in the World Health Organization’s watch group because of its high resistance potential, driven primarily by the accumulation of point mutations in *gyr(A)* and *par(C)* genes. Levofloxacin-resistant GBS remains relatively uncommon, with estimated resistance rates of 8.6%^16^ and up to 5% in Brazil^15^, which is consistent with the rate of 5.4% observed in our study. Nonetheless, we noted significant year-to-year variations. In two years (2021 and 2024), no resistant isolates were detected; however, a peak was observed in 2023. Given that fluoroquinolone resistance progresses from low to high level through stepwise accumulation of mutations^17^, the observed pattern is likely linked to prior fluoroquinolone exposure in patients, which could promote the stepwise development of resistance to levofloxacin.

Resistance to tetracyclines is estimated to be 80.1%^16^, primarily mediated by the *tet(M)* gene and less frequently by *tet(O)*. Although tetracyclines are not used to treat GBS infections, some studies suggest that the acquisition of *tet(M)* contributes to the stepwise selection and dissemination of virulent GBS lineages^18^. Data from our five-year study consistently revealed a high tetracycline resistance rate of 82.3%, driven primarily by the presence of *tet(M)* (93.9%), followed by *tet(O)* (6.1%).

Despite the significant findings obtained from this single-center study of a high-risk pregnancy population in northeast Brazil, there are limitations in drawing certain conclusions. As mentioned previously, the culture method employed showed low sensitivity, thereby limiting isolate recovery. Additionally, owing to convenience sampling for genotyping, only few anovaginal isolates were obtained in 2020, 2022, and 2023. Consequently, these isolates were not included in the study, resulting in a limited evaluation of their molecular epidemiology. Additionally, there were no patient data correlating with prior use of antibiotics.

In conclusion, our findings underscore the dynamics of GBS colonization among high-risk pregnant women in northeast Brazil, showing a reduced colonization rate in the local population during the post-pandemic period. Although penicillin and ampicillin remain effective for IAP, continuous surveillance of antibiotic resistance in GBS is essential to guide IAP and treatment strategies. Furthermore, the observed increasing macrolide resistance pattern in our setting over the past five years could potentially compromise clindamycin efficacy, particularly through its association with the presence of *erm* genes.

## Data Availability

Research data is only available upon request.
